# Cardiovascular and cancer mortality in relation to dietary polychlorinated biphenyls and marine polyunsaturated fatty acids: a nutritional‐toxicological aspect of fish consumption

**DOI:** 10.1111/joim.12995

**Published:** 2019-11-08

**Authors:** C. Donat‐Vargas, A. Bellavia, M. Berglund, A. Glynn, A. Wolk, A. Åkesson

**Affiliations:** ^1^ Unit of Cardiovascular and Nutritional Epidemiology Institute of Environmental Medicine Karolinska Institutet Stockholm Sweden; ^2^ Department of Preventive Medicine and Public Health School of Medicine Universidad Autónoma de Madrid, CEI UAM+CSIC Madrid Spain; ^3^ Department of Environmental Health Harvard T.H. Chan School of Public Health Boston MA USA; ^4^ Department of Biomedical Sciences and Veterinary Public Health Swedish University of Agricultural Sciences (SLU) Uppsala Sweden

**Keywords:** all‐cause mortality, long‐chain n‐3 polyunsaturated fatty acids (LC n‐3 PUFAs), nutritional epidemiology, polychlorinated biphenyls (PCBs), specific mortality

## Abstract

**Background:**

Co‐exposure to environmental contaminants present in fish could mitigate the beneficial effects of fish consumption and possibly explain the lack of association observed for mortality in some geographical regions.

**Objective:**

To assess the independent associations of dietary exposure to polychlorinated biphenyls (PCBs) and long‐chain omega‐3 fish fatty acids intake with cardiovascular and cancer mortality.

**Methods:**

We used the prospective population‐based Swedish Mammography Cohort and the Cohort of Swedish Men comprising 32 952 women and 36 545 men, free from cancer, cardiovascular disease and diabetes at baseline in 1998. Validated estimates of dietary PCBs and long‐chain omega‐3 fish fatty acids [i.e. eicosapentaenoic acid (EPA) and docosahexaenoic acid (DHA)] intake were obtained via a food frequency questionnaire at baseline. Information on death was ascertained through register linkage.

**Results:**

During a mean follow‐up of 15.5 years, we ascertained 16 776 deaths. We observed for cardiovascular mortality, comparing extreme quintiles in multivariable models mutually adjusted for PCBs and EPA‐DHA, dose‐dependent associations for dietary PCB exposure, hazard ratio (HR) 1.31 (CI 95%: 1.08 to 1.57; *P*‐trend 0.005) and for dietary EPA‐DHA intake, HR 0.79 (CI 95%: 0.66 to 0.95; *P*‐trend 0.041). For cancer mortality, no clear associations were discerned.

**Conclusion:**

The beneficial effect of fish consumption on the cardiovascular system seems compromised by co‐exposure to PCBs – one likely explanation for the inconsistent associations observed between fish consumption and mortality.

## Graphical abstract

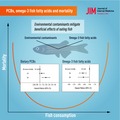

## Introduction

Epidemiological studies elucidating the beneficial effects of fish consumption in relation to mortality have not revealed a clear picture. While Asian studies generally reported inverse associations 1, 2, some Western studies found no association 3, 4 or even a higher risk of mortality associated with high fish consumption 5, 6, 7. This heterogeneity was reflected in the most recent meta‐analysis (including 14 prospective observational studies) 8, which, overall, reported no meaningful association between fish consumption and mortality. However, in the analysis by region, while Asian studies revealed a linear inverse association, Western studies advocated a nearly U‐shaped association, with a nadir at fish consumption of ~20 g day^−1^ for both total and cardiovascular (CVD) mortality 8. This summarized evidence agrees with our previous results indicating a U‐shaped association between fish consumption and mortality in a large Swedish population‐based cohort 5, which was the driving force behind the present work.

Fatty fish is the exclusive natural food source of long‐chain ω‐3 polyunsaturated fatty acids (ω‐3 PUFAs), mainly eicosapentaenoic acid (EPA) and docosapentaenoic acid (DHA) 9. The beneficial role of ω‐3 PUFAs on blood lipids, cardiac electrophysiology, endothelial function and blood pressure has been consistently reported 10. On the other hand, polychlorinated biphenyls (PCBs), dioxin‐like compounds (DL‐Cs) and methylmercury (MeHg) are identified as the most critical contaminants bioaccumulating in fish 11. In addition to be classified as carcinogenic 12, PCBs are implicated in CVD development 13, 14, 15, 16, 17 and in its risk factors, such as hypertension 18, obesity and diabetes 19. Biomarkers of DL‐C, which include dioxin‐like PCBs, were associated with higher rate of mortality in US populations 20, 21, 22. However, any potential confounding by a healthy diet, fish or EPA‐DHA intakes was not considered. MeHg was not associated with CVD in a large US population 23, but in a Nordic population after adjustment for EPA‐DHA 24.

We hypothesize that high exposure to certain environmental contaminants present in fish, particularly PCBs, could mitigate the beneficial effects mainly by EPA‐DHA 25 – being a feasible explanation for the observed higher risk associated with high fish consumption in Western countries. Thus, the purpose of the present study was to elucidate the independent associations between dietary PCB exposure and EPA‐DHA intake in relation to CVD‐ and cancer‐specific mortality in two large population‐based cohorts of men and women, accounting for MeHg exposure.

## Methods

### Study population

The present study included participants from the Swedish Infrastructure for Medical Population‐based Life‐course and Environmental Research (SIMPLER), specifically the Swedish Mammography Cohort (SMC) and the Cohort of Swedish Men (COSM). Participants from the SMC were recruited between 1987 and 1990 and included women born between 1914 and 1948 and residing in central Sweden (*n* = 90 303) and 74% responded. In 1997, a detailed questionnaire was sent to all participants still alive and living in the study area and 39 227 responded (70%). Likewise, participants from the COSM were recruited 1997 and included men born between 1918 and 1952 and residing in central Sweden (48 850 responded, 49%). The completed 1997 questionnaires were used for the baseline exposure assessment in both men and women.

**Figure 1:**
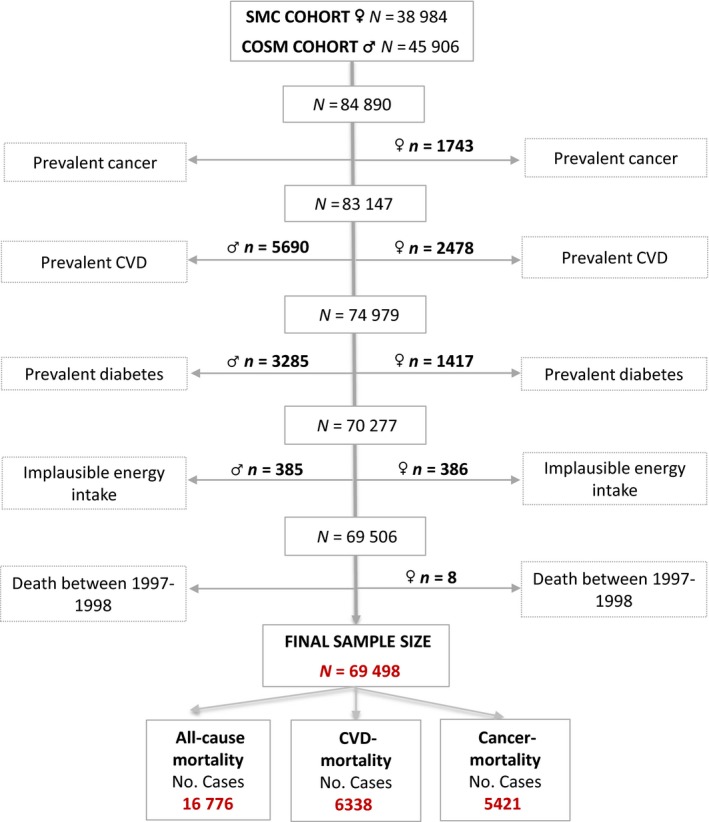
Flow chart.

For the present study, we excluded individuals who had a previous diagnosis of cancer (*n* = 1743 ♀ and *n* = 2592 ♂), CVD (*n* = 2478 ♀ and *n* = 5690 ♂) or/and type 2 diabetes (*n* = 1417 ♀ and *n* = 3285 ♂), and those with extreme energy intakes (±3 standard deviations of mean log‐transformed energy intake; *n* = 386 ♀ and *n* = 385 ♂). We also excluded those women (*n* = 8) who died between the administration of the SMC baseline questionnaire (September 1997) and the start of the follow‐up (1 January 1998). Hence, the final study population involved 32 952 women and 36 546 men (Fig. 1). The study was approved by the Regional Ethical Review Board at Karolinska Institutet (Stockholm, Sweden), and all participants gave their informed consent.

### Assessment of dietary PCB, long‐chain ω‐3 fatty acids and covariates

A validated semi‐quantitative 96‐item food frequency questionnaire (FFQ) was used to assess average intake, over the preceding year, of fish and other foods. Participants could choose from eight predefined consumption categories (never, 1–3/month, 1–2/week, 3–4/week, 5–6/week, 1/day, 2/day and 3/day).

Daily dietary intakes of PCBs (ng day^−1^), MeHg (µg day^−1^) and the marine ω‐3 PUFAs, EPA‐DHA (g day^−1^), were estimated by multiplying their average concentration in various foods (obtained from the Swedish National Food Agency’s food control and monitoring programmes, Swedish Environmental Protection Agency and the Swedish food composition database) with the respective consumption frequency and age and sex‐specific portion size. The dietary exposure to PCBs was based on concentrations of the PCB‐153 congener 26, which is the most abundant congener in food and therefore a very good indicator of both total PCBs and dioxin‐like PCBs in food as well as in human serum 11, 27. Fish, including shellfish, contributed to about 2/3 of the total dietary PCB exposure in the cohorts. Estimation of dietary MeHg exposure was based on fish only as it is practically the only source of dietary MeHg. In order to ensure that the associations were independent of the total energy intake, estimated dietary PCB, MeHg and EPA‐DHA intakes were adjusted for total daily energy intake using the residual‐regression method 28. The Spearman correlation coefficients (ρ) were 0.91 between quintiles of EPA‐DHA and PCBs, 0.62 between EPA‐DHA and MeHg and 0.64 between PCBs and MeHg.

The FFQ‐based dietary estimate of PCB exposure has been extensively validated against serum concentrations of PCB congeners (PCB‐118, PCB‐138, PCB‐153. PCB‐156 and PCB‐180) in a representative subsample of the SMC cohort ranged from ρ 0.30 to 0.58] 26. Likewise, the FFQ‐based dietary intake of EPA‐DHA has also been validated against adipose tissue concentrations of EPA and DHA [*r* = 0.32 and 0.48, respectively, for concurrent exposure, and ρ = 0.21 and 0.33, respectively, for past exposure (6 years prior to sampling)] 29. FFQ‐estimated fish consumption has been shown to correlate well with hair mercury concentrations in a Swedish population (ρ = 0.75; *P* < 0.001) 30.

As a measure of a healthy dietary pattern, the FFQ was also used to create a modified Mediterranean diet score (ranging from 0 [lowest adherence] to 8 [highest adherence]), based on intake of seven different food groups [fruits and vegetables, legumes and nuts, whole grain/fibre‐rich foods, fermented dairy foods, fish, red and processed meat (as a negative component), and olive/rapeseed oil] and alcohol in moderation (10–30 g day^−1^ for men and 5–15 g day^−1^ for women), as described in detail elsewhere 31. The self‐administered questionnaire inquired about education, cigarette smoking, weight and weight loss, waist circumference, height, physical activity, family history of myocardial infarction, drugs and dietary supplement use, and alcohol consumption.

### Case ascertainment

Information on death and causes of death was ascertained through linkage to the Swedish Cause of Death Register at the National Board of Health and Welfare. In Sweden, 93% of deaths are reported within 10 days, and 100% are reported within 1 month 32. The cause of death was coded using the International Classification of Diseases, 10th Revision (ICD‐10).Cause‐specific mortality was ascertained for CVD (codes I00‐I78) and cancer (codes C00‐C97).

### Statistical analyses

Person‐time of follow‐up was calculated for each participant from 1 January 1998 until death or end of follow‐up in 31 December 2014 whichever occurred first. Associations between dietary exposures and CVD‐ and cancer‐specific mortality were assessed using Cox proportional hazards regression, with attained age as the time scale, reporting hazard ratios (HR) and 95% confidence intervals (CI). All analysis were stratified by sex (i.e. by cohort SMC/COSM).

Associations were evaluated using cohort‐specific quintiles of the exposures. All models were adjusted for the following baseline potential confounders: attained age (years), education level (≤12 or >12 years), waist circumference (♀ <80, 80–87, ≥88 cm; ♂ <94, 94–102, >102 cm), hypertension (yes/no), hypercholesterolaemia (yes/no), weight loss >5kg within 1 year (yes/no), leisure‐time inactivity (≤2 or >2 h day^−1^) and daily walking/cycling (≤40 or >40 min day^−1^), family history of myocardial infarction before the age of 60 years (yes/no), smoking status (current, former, never), use of aspirin (yes/no), energy intake (continuous, kcal day^−1^), Mediterranean diet score, dietary MeHg exposure (quintiles) and, in women, also for parity and use of hormone replacement therapy (yes/no). In an additional model, we further adjusted for either dietary exposure of EPA‐DHA or PCB in quintiles, as appropriate. Given that frying of fish may depress the beneficial effects of fish consumption 2, we also took the cooking method of fish into consideration. Because additional adjustment for categories of fried fish consumption (five groups; ≤1, 2, 3–4, 5, ≥6 servings month^−1^) did not affect any estimation these results are not shown.

We fitted the multivariable‐adjusted models including the missing values – below 3% for all covariates with the exception of waist circumference (15%) and daily walking/cycling (14%) – in a separate category. The adequacy of proportional hazards assumption was checked by using Schoenfeld residuals, and no evidence of departure from this assumption was observed for the main exposures.

To test for linear trends (*P*‐trend) across increasing categories of PCB/EPA‐DHA exposures, the median concentration within each quintile and category of sex was included and treated as a continuous variable in the model. Likewise, restricted cubic splines with 3 knots of the distribution (at the 10th, 50th and 90th percentiles of the distribution) were used to flexibly model the dose‐response associations and allow for non‐linearity. Furthermore, all spline models were replicated to evaluate associations in terms of survival time, using quantile regression for survival data to estimate differences in median age at death over levels of dietary PCB exposure and EPA‐DHA intake 33. Lastly, the potential interactions between dietary PCB and EPA‐DHA exposures was tested using the likelihood ratio test, comparing Cox models with and without interaction term (for interaction on the multiplicative scale) and using survival percentiles comparing quantile regression models with and without interaction term (for interaction on the additive scale). All analyses were run in Stata version 14.0 (StataCorp LP, College Station, TX, USA), with statistical significance set at the two‐sided 0.05 level.

## Results

During a mean follow‐up of 15.5 years (person‐time‐at risk 1 073 479 years), 16 776 total deaths (6338 due to CVD and 5421 to cancer) were ascertained (Fig. 1). The average age (years) at death was 81 (±9) for women and 78 (±9) for men. Table 1 depicts age‐standardized baseline characteristics of the study population by quintiles of dietary PCB exposure. The median (5th‐95th percentiles) of dietary PCB exposure was 165 (70–370) ng day^−1^ in women and 231 (87–531) ng day^−1^ in men. The corresponding results for EPA‐DHA were 288 (87–689) mg day^−1^ and 395 (99–971) mg day^−1^, respectively. Both women and men in the highest quintile of dietary PCB exposure had a higher dietary intake of both EPA‐DHA and higher exposure to MeHg compared with those in the lowest quintile. The other baseline characteristics were generally similar between categories of dietary PCB exposure.

**Table 1:** Age‐standardized baseline characteristics of 32 952 women and 36 545 men by quintiles of dietary PCB exposure

Baseline characteristics	PCB exposure (quintiles)									
	Women					Men				
	Q1	Q2	Q3	Q4	Q5	Q1	Q2	Q3	Q4	Q5
No. subjects	6591	6590	6591	6590	6590	7310	7309	7309	7309	7309
Dietary PCB exposure (ng day^−1^)	87 ± 24	135 ± 9	165 ± 9	206 ± 18	356 ± 182	112 ± 32	187 ± 15	231 ± 12	282 ± 22	512 ± 285
EPA/DHA intake (mg/d)	132 ± 60	224 ± 48	286 ± 46	370 ± 60	646 ± 36	161 ± 81	300 ± 64	393 ± 64	501 ± 76	921 ± 59
Dietary methylmercury exposure (µg day^−1^)	0.64 ± 0.48	0.88 ± 0.48	1.13 ± 0.48	1.50 ± 0.66	2.36 ± 2.61	0.74 ± 0.62	0.99 ± 0.55	1.26 ± 0.54	1.76 ± 0.73	2.62 ± 2.28
Age (years)	61 ± 9	61 ± 9	60 ± 9	61 ± 9	64 ± 9	57 ± 10	59 ± 10	58 ± 9	58 ± 9	61 ± 9
Postsecondary education >12 years (%)	18	21	21	20	19	16	16	19	21	20
Family history of MI before age of 60 years (%)	16	16	16	17	18	15	15	15	16	16
Waist circumference, cm										
women: <80 men: <94	39	39	38	39	36	3	3	3	2	3
women: 80–87 men: 94–102	31	32	32	31	32	16	16	17	15	15
women: ≥88 men: >102	31	29	30	29	32	81	81	81	82	82
Weight loss >5 kg within 1 year (%)	68	67	69	69	70	40	38	38	40	42
History of hypertension (%)	18	18	18	19	20	13	14	13	14	15
History of cholesterol (%)	7	7	7	7	8	6	7	8	9	8
Leisure‐time inactivity >2 h day^−1^, (%)	58	56	56	58	58	64	63	64	65	65
Walking/cycling >40 min day^−1^, (%)	31	32	31	31	32	26	26	26	25	27
Smoking (%)										
Former	22	22	24	24	24	35	38	38	38	39
Current	25	23	22	24	25	26	24	24	24	26
Use of aspirin (%)	41	44	45	44	42	32	33	33	32	33
Energy intake (kcal day^−1^)	1762 ± 568	1839 ± 507	1708 ± 441	1673 ± 535	1731 ± 534	2663 ± 878	2920 ± 868	2773 ± 730	2491 ± 748	2719 ± 874
Mediterranean diet adherence (9‐score)	4 ± 2	4 ± 2	4 ± 2	4 ± 2	5 ± 2	3 ± 2	4 ± 2	4 ± 2	4 ± 1	4 ± 1
Fried fish (serving month^−1^)	2.5 ± 2.1	2.8 ± 2.2	3.0 ± 2.3	3.1 ± 2.4	3.6 ± 2.8	2.7 ± 2.3	3.1 ± 2.2	3.3 ± 2.5	3.5 ± 2.3	4.1 ± 2.9
Parity										
Nulliparous	10	9	9	9	10					
1–2 child	57	58	59	58	56					
>2 child	33	33	33	33	34					
Hormone replacement therapy (%)	50	51	52	52	52					

**Figure 2:**
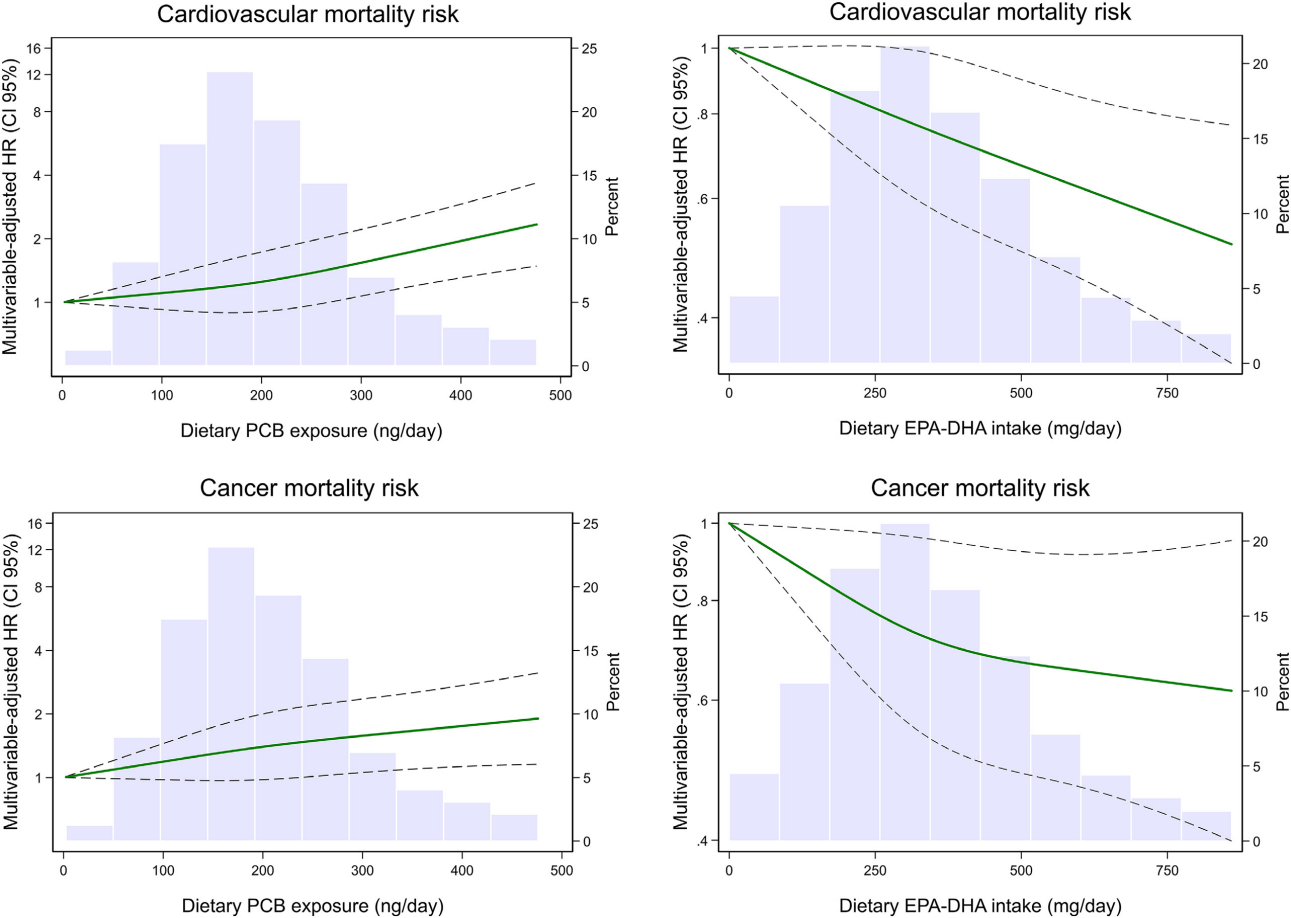
Cohort‐stratified hazard ratios (HR) of cardiovascular and cancer death as a function of dietary PCB and EPA‐DHA intakes. Data were fitted using Cox proportional hazard regression evaluated with restricted cubic splines with three knots of the distribution (at the 10th, 50th and 90th percentiles of the distribution) Participants with an exposure above the 95th percentile are not included. Dashed lines represent 95% CIs. The histograms show the distributions of dietary PCB exposure and dietary EPA/DHA intake. Models adjusted for attained age (years), education level (≤12 or >12 years), waist circumference (<80, 80–87, ≥88 cm), hypertension (yes/no), hypercholesterolaemia (yes/no), weight loss >5 kg within 1 year (yes/no), leisure‐time inactivity (≤2 or >2 h day^−1^) and daily walking/cycling (≤40 or >40 min day^−1^), family history of myocardial infarction before the age of 60 years (yes/no), smoking status (current, former, never), use of aspirin (yes/no), energy intake (continuous, kcal day^−1^), Mediterranean diet (9‐score), parity (0, 1–2, ≥ 3 child), use of hormone replacement therapy (yes/no) and dietary methylmercury exposure (quintiles) and, respectively, for EPA‐DHA intake (quintiles) or dietary PCB exposure (quintiles).

After multivariable‐adjustment and taking into account EPA‐DHA intake in the model, the highest PCB‐exposed participants (women and men) had a significant 20% higher risk of death than those least exposed (HR 1.20; CI 95%:1.07 to 1.34; *P*‐trend = 0.001). The corresponding HR for CVD‐specific deaths was 1.31 (CI 95%:1.08 to 1.57; *P*‐trend = 0.005). For EPA‐DHA intake (taking into account dietary PCB exposure), the HR for all death was 0.88 (CI 95%: 0.78 to 0.98; *P*‐trend = 0.23) and for CVD‐specific death 0.79 (CI 95%: 0.66 to 0.95; *P*‐trend = 0.041) (Table 2), Fig. 2).

**Table 2:** Cohort‐stratified hazard ratios (HR) of all‐cause and cause‐specific mortality according to quintiles of dietary PCB and EPA‐DHA intakes in 69 497 women and men (1998–2014)

	Dietary PCB exposure (ng day^−1^)						Dietary EPA‐DHA intake (mg day^−1^)					
	Q1	Q2	Q3	Q4	Q5	*P*‐trend	Q1	Q2	Q3	Q4	Q5	*P*‐trend
Median (ng day^−1^) ***♀***	93	136	164	203	305		133	223	286	364	551	
Median (ng day^−1^) ***♂***	113	188	231	278	496		157	296	394	497	780	
Mortality												
All‐cause												
Person‐years	213 814	214 384	218 672	218 402	208 207		211 736	216 051	218 412	217 146	210 134	
No of cases	3424	3381	2837	2864	4270		3694	3200	2857	3025	4000	
Age and gender‐adjusted HR (95% CI)	1.00	0.92 (0.87, 0.96)	0.87 (0.82, 0.91)	0.86 (0.82, 0.91)	0.98 (0.93, 1.02)	0.607	1.00	0.87 (0.83, 0.91)	0.83 (0.79, 0.88)	0.85 (0.81, 0.89)	0.95 (0.91, 0.99)	0.692
Multivariable ‐adjusted HR (95% CI)^a^	1.00	0.97 (0.93, 1.02)	0.95 (0.90, 1.00)	0.95 (0.90, 1.01)	1.08 (1.02, 1.14)	<0.001	1.00	0.90 (0.86, 0.95)	0.91 (0.86, 0.96)	0.92 (0.87, 0.97)	1.02 (0.96, 1.08)	0.025
Multivariable‐adjusted HR (95% CI)^b^	1.00	1.06 (0.99, 1.12)	1.06 (0.98, 1.15)	1.07 (0.97, 1.18)	1.20 (1.07, 1.34)	0.001	1.00	0.87 (0.82, 0.93)	0.87 (0.80, 0.94)	0.86 (0.78, 0.94)	0.88 (0.78, 0.98)	0.232
Cardiovascular												
No of cases	1332	1268	1020	1071	1647		1439	1212	1054	1126	1507	
Age and gender ‐adjusted HR (95% CI)	1.00	0.89 (0.82, 0.96)	0.83 (0.76, 0.90)	0.85 (0.78, 0.92)	0.94 (0.88, 1.01)	0.708	1.00	0.87 (0.80, 0.94)	0.83 (0.76, 0.90)	0.84 (0.78, 0.91)	0.92 (0.85, 0.99)	0.165
Multivariable‐adjusted HR (95% CI)^a^	1.00	0.95 (0.88, 1.03)	0.93 (0.85, 1.01)	0.96 (0.88, 1.06)	1.07 (0.97, 1.17)	0.024	1.00	0.91 (0.84, 0.98)	0.91 (0.83, 0.99)	0.93 (0.85, 1.02)	1.00 (0.91, 1.09)	0.471
Multivariable‐adjusted HR (95% CI)^b^	1.00	1.02 (0.92, 1.13)	1.04 (0.91, 1.19)	1.13 (0.97, 1.33)	1.31 (1.08, 1.57)	0.005	1.00	0.89 (0.80, 0.99)	0.87 (0.76, 0.99)	0.82 (0.70, 0.95)	0.79 (0.66, 0.95)	0.041
Cancer												
No of cases	1051	1115	981	976	1298		1132	1047	968	1020	1254	
Age and gender ‐adjusted HR (95% CI)	1.00	0.97 (0.89, 1.06)	0.92 (0.84, 1.01)	0.91 (0.84, 0.99)	1.00 (0.93, 1.09)	0.691	1.00	0.90 (0.83, 0.97)	0.86 (0.79, 0.94)	0.88 (0.81, 0.96)	0.97 (0.90, 1.05)	0.919
Multivariable ‐adjusted HR (95% CI)^a^	1.00	1.00 (0.92, 1.10)	0.97 (0.88, 1.06)	0.95 (0.86, 1.05)	1.05 (0.94, 1.16)	0.282	1.00	0.91 (0.84, 1.00)	0.90 (0.82, 0.99)	0.92 (0.83, 1.01)	0.99 (0.90, 1.10)	0.553
Multivariable ‐adjusted HR (95% CI)^b^	1.00	1.10 (0.98, 1.23)	1.09 (0.94, 1.25)	1.05 (0.89, 1.24)	1.10 (0.90, 1.35)	0.444	1.00	0.87 (0.77, 0.97)	0.85 (0.74, 0.98)	0.88 (0.75, 1.04)	0.93 (0.76, 1.13)	0.635

a Adjusted for attained age (years), gender, education level (≤12 or >12 years), waist circumference (<80, 80–87, ≥88 cm), hypertension (yes/no), hypercholesterolaemia (yes/no), weight loss >5 kg within 1 year (yes/no), leisure‐time inactivity (≤2 or >2 h day^−1^) and daily walking/cycling (≤40 or >40 min day^−1^), family history of myocardial infarction before the age of 60 years (yes/no), smoking status (current, former, never), use of aspirin (yes/no), energy intake (continuous, kcal day^−1^), Mediterranean diet (9‐score), parity (0, 1–2, ≥ 3 child), use of hormone replacement therapy (yes/no) and dietary methylmercury exposure (quintiles).

b Additional adjusted for EPA‐DHA intake (quintiles) or dietary PCB exposure (quintiles).

Dietary PCB was not associated with a higher cancer‐specific mortality (HR 1.10; CI 95%:0.90 to 1.35; *P*‐trend = 0.4). EPA‐DHA intakes were, however, significantly associated with lower cancer mortality up to the mid‐quintile but not at higher intakes (Table 2). There was no evidence of interactions by sex or between dietary PCB and EPA‐DHA exposures. We observed no associations between dietary MeHg exposure and mortality (Table S1).

**Figure 3:**
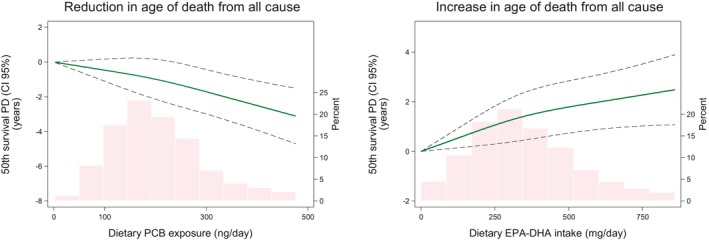
50th survival percentile difference (PD) (i.e. difference in median age at death), according to dietary PCB and EPA‐DHA exposures. Data were fitted using quantile regression for survival data evaluated with restricted cubic splines with three knots of the distribution (at the 10th, 50th and 90th percentiles of the distribution) Participants with an exposure above the 95th percentile are not included. Dashed lines represent 95% CIs. The histograms show the distributions of dietary PCB exposure and dietary EPA/DHA intake. Models adjusted for attained age (years), cohort, education level (≤12 or >12 years), waist circumference (<80, 80–87, ≥88 cm), hypertension (yes/no), hypercholesterolaemia (yes/no), weight loss >5 kg within 1 year (yes/no), leisure‐time inactivity (≤2 or >2 h day^−1^) and daily walking/cycling (≤40 or >40 min day^−1^), family history of myocardial infarction before the age of 60 years (yes/no), smoking status (current, former, never), use of aspirin (yes/no), energy intake (continuous, kcal day^−1^), Mediterranean diet (9‐score), parity (0, 1–2, ≥ 3 child), use of hormone replacement therapy (yes/no) and dietary methylmercury exposure (quintiles) and respectively for EPA‐DHA intake (quintiles) or dietary PCB exposure (quintiles).

Compared to the lowest quintile of dietary PCB exposure, the highest quintile was associated with a lower median age at death of approximately 9 months and, similarly, the highest quintile of EPA‐DHA intake was associated with a higher median age at death of approximately 6 months (data not shown). The corresponding shapes of the difference in median age at death over levels of dietary PCB and EPA‐DHA exposures are represented by spline models evaluating associations in terms of survival time (Fig. 3).

## Discussion

In these two large population‐based cohorts with long follow‐up, dietary PCB exposure was dose‐dependently associated with increased CVD mortality, whereas the intake of EPA‐DHA was dose‐dependently associated with lower CVD mortality. No association was discerned between PCB exposure and cancer mortality, while some indication of a u‐shaped association was observed for the EPA‐DHA intake. These results suggest that the high content of PCBs in certain fish species could explain, at least in part, the increased mortality observed at high fish consumption (right end of the U‐shaped association) in certain areas of the world.

Several mechanisms related to the pathogenesis of CVD and other metabolic disorders may be triggered by PCBs. Experimental evidence reveals that PCBs induce chronic inflammation and dysfunction in the vascular endothelium, via expression of several inflammatory markers 34, 35, 36 and increasing cellular oxidative stress 37. Likewise, some of these effects are suggested to be induced through epigenetic regulation such as histone modifications 36 or altered expression of miRNAs, which is associated with cardiac injury and inflammation 38. In accordance, a growing number of observational studies in general populations have linked PCB exposure to different cardiometabolic risk factors including hypertension 18, type 2 diabetes and obesity 19, as well as to CVD 13, 15, 16, 39, 40. This human evidence is supported by animal data 37, 41, 42.

Early cohort studies with high PCB‐exposed workers (through inhalation or skin absorption) reported elevated mortality from CVD 43, 44 and less consistent from cancers 45, 46, 47, 48. In the general population, mortality risk in relation to background‐PCB exposure has only been addressed by three American prospective studies. Biomarkers of DL‐Cs were associated with a significant 19 % increased risk of total mortality and, albeit non‐significant, comparable risk of CVD and cancer mortality 21. The PCBs were, however, not clearly associated with increased either total or cause‐specific mortality in the other two studies 20, 22. These studies did not account for fish, ω‐3 PUFAs intakes or a healthy diet.

There is scarce evidence on PCB‐carcinogenicity among nonoccupationally exposed populations. Although biologically plausible, the PCB classification into group 1 by the IARC was based on the risk of melanoma mainly in workers highly exposed to PCBs, as the evidence on both non‐Hodgkin lymphoma and breast cancers is limited and for other cancers sites inconclusive 49. While we previously observed association between dietary PCB exposure and incidence of malignant melanoma and high‐grade and fatal prostate cancer in our cohort 50, there were mainly null‐associations in relation to the development of female hormone‐related cancers 51, 52. Thus, the null association between dietary PCB exposure and cancer mortality it is not surprising.

In contrast, EPA‐DHA, which have an essential function for the metabolism, are identified as the major profit of ingesting fish‐derived nutrients. The claimed cardio‐protective effects of EPA‐DHA appear not to be through a single mode of action but to a synergism between multiple mechanisms involving the improvement of inflammation, endothelial function, arterial compliance (elasticity) 53 as well as its capacity to diminish the oxidative stress 54. Scientific research from randomized controlled trials (RCT) on EPA‐DHA supplementation has shown that EPA‐DHA also decrease triglycerides, increase HDL cholesterol and LDL particle size (which may be less atherogenic), reduce heart rate and blood pressure and inhibit platelet function 55, 56. Likewise, there is a biological rationale for the protective role of EPA and DHA in the genesis of cancer 57, 58, 59.

Our findings are, however, in keeping with a meta‐analysis 60 involving over 30 thousand deaths events from 11 prospective studies including general population from USA and Asia, where both dietary and circulating ω‐3 PUFAs showed to be significantly associated with reduced risk of all‐cause mortality. Whether these contrasting findings to the null effects observed in ω‐3 PUFAs supplemental RCTs 61, indicate confounding by other dietary or non‐dietary factors, or that other components of fish are responsible for the observed associations, is not known. Regarding our results, the EPA‐DHA intake displayed a u‐shaped tendency for cancer mortality, while for CVD mortality there was a clear dose‐dependent protective association.

The regional differences observed between fish consumption and mortality 8, 62 could partly be due to geographical variation in type of seafood consumed – with differences in nutrient and contaminant content – and in local contamination. Japan/Korea/Philippines followed by the Nordic‐Baltic countries and South‐East Asia have the largest seafood consumption 8. Japan has the highest EPA‐DHA intake followed by Nordic countries according to an exposure assessment from the Global Environment Monitoring System 63. In the Nordic‐Baltic countries, while pelagic marine fishes are the most consumed, the intake of crustacean and cephalopods is limited. In Asiatic countries, however, the crustacean and cephalopods contribute to more than 40% to the total seafood consumption followed by freshwater fishes. Because pelagic marine fishes have the highest fat content, they also have the highest concentration of fat‐soluble nutrients (e.g. EPA‐DHA and vitamin D) as well as lipophilic contaminants such as PCBs and DL‐Cs. Freshwater fish can be high in PCBs and DL‐Cs depending on the industrial contamination. On the other hand, high MeHg is mainly found in those fish species being at the top of the aquatic trophic chain (such as shark, tuna or swordfish) or freshwater predatory fishes (such as pike and pike perch in Sweden). Thus, even at a similar amount of seafood consumption, the exposure profile to seafood‐derived contaminants will vary depending on the type of fish. While Japan seems to have higher MeHg and lower PCB intakes, the Nordic–Baltic countries apparently have lower MeHg and higher PCB intakes 8. Thus, it may be more be difficult to reach the desired intake levels for EPA‐DHA in this population, without exceeding the potential negative consequences of the dietary PCB exposure.

Fish can be also a potential source of human exposure to other toxic environmental contaminants such as polychlorinated dibenzofurans, polycyclic aromatic hydrocarbons (mostly smoked fish), polybrominated diphenyl ethers, chlorinated pesticides, or per‐ and polyfluorinated alkyl substances 25, 64. Future studies further considering the overall impact of the co‐exposure of these different contaminants present in fish is necessary to be able to make a rigorous real assessment of the risk‐benefit of fish consumption.

Strengths of the present study include its large sample size providing statistical power and the prospective population‐based design, which avoids reverse causation bias. The availability of information on demographics, risk factors and lifestyle prospectively collected allowed us to adjust for multiple covariates, minimizing confounding. In addition, the practically complete follow‐up through computerized linkage to the Swedish Cause of Death Register minimized possible bias due to differential loss to follow‐up. Importantly, dietary PCBs and EPA‐DHA exposures were validated against serum and adipose tissue concentrations, respectively. Adequate validity is essential to ensure that the potential recall bias and measurements errors derived from self‐reported dietary intakes are as low as possible.

Limitations should be reflected. Exposures were measured at baseline, assuming that dietary habits remained the same over the entire period. Dietary variations over time would increase exposure misclassification during follow‐up. Also, misreporting of dietary intake (measurement error) cannot be ruled out, contributing to the exposure misclassification. The exposure misclassification is most likely nondifferential, potentially leading to attenuated risk estimates, although risk estimates biased away from the null cannot be excluded. The observational design cannot ignore residual confounding by unknown or unmeasured factors including other contaminants also present in fish. Yet, results were robust to adjustment for multiple major risk factors. Due to strong confounding, the associations were only observed after mutual adjustments for PCBs and EPA‐DHA, raising the issue with collinearity. It is known that multicollinearity can cause unstable estimates and inaccurate variances which affects confidence intervals and hypothesis tests. There were, however, no indications of inflated confidence intervals or unstable risk estimates in the present study, implying no major effect of collinearity. This conclusion was further supported by recent simulations indicating that for models properly specified, collinearity (unless extreme; ρ > 0.999) does not have to induce any measurable bias 65. Finally, we could not distinguish between dioxin‐like and nondioxin‐like PCBs and either them from other contaminants present in the same foods such as polychlorinated p‐dioxins and furans, which have also been associated with CVD.

## Conclusion

In this prospective assessment in Swedish middle‐aged and elderly men and women, high dietary PCB exposure was associated with increased CVD mortality and EPA‐DHA intake with decreased CVD mortality. This counteraction may explain the increased mortality observed at high fish consumption (right end of the U‐shape) in certain areas of the world and the net benefit of fish consumption may depend on the PCB contamination.

## Conflict of interest statement

None of the authors has any competing financial interests to declare.

## Supporting information


**Table S1.** Cohort‐stratified hazard ratios (HR) of all‐cause and cause‐specific mortality according to quintiles of dietary methylmercury exposure in 69,497 women and men (1998‐2014).
