# Supplementary material for: Cardiovascular and cancer mortality in relation to dietary polychlorinated biphenyls and marine polyunsaturated fatty acids: a nutritional‐toxicological aspect of fish consumption
Source: J Intern Med. 2019 Nov 8;287(2):197–209. doi: 10.1111/joim.12995 (PMC7003855; doi:10.1111/joim.12995)
Supplement: Supplementary file 1 — Table S1. Cohort‐stratified hazard ratios (HR) of all‐cause and cause‐specific mortality according to quintiles of dietary methylmercury exposure in 69,497 women and men (1998‐2014). [file JOIM-287-197-s001.docx]

| **Table S1** Cohort-stratified hazard ratios (HR) of all-cause and cause-specific mortality according to quintiles of dietary methylmercury exposure in 69,497 women and men (1998-2014) | | | | | | |
| --- | --- | --- | --- | --- | --- | --- |
|  | **Dietary methylmercury exposure (µg/day)** | | | | | |
|  | **Q1** | **Q2** | **Q3** | **Q4** | **Q5** | P trend |
| Median (ng/day) ***♀*** | 0.55 | 0.78 | 1.02 | 1.43 | 1.82 |  |
| Median (ng/day) ***♂*** | 0.61 | 0.87 | 1.12 | 1.66 | 2.21 |  |
| **MORTALITY** |  |  |  |  |  |  |
| **All-cause** |  |  |  |  |  |  |
| *Person-years* | 213,167 | 216,605 | 216,751 | 216,247 | 210,708 |  |
| No of cases | 3,487 | 3,124 | 3,069 | 3,215 | 3,881 |  |
| Age-adjusted HR  (95% CI) | 1.00 | 0.88  (0.84, 0.93) | 0.90  (0.86, 0.95) | 0.87  (0.83, 0.91) | 0.93  (0.89, 0.97) | 0.042 |
| Multivariable-adjusted  HR (95% CI) | 1.00 | 0.96  (0.91, 1.01) | 1.00  (0.95, 1.06) | 0.98  (0.92, 1.04) | 0.99  (0.93, 1.06) | 0.826 |
| **Cardiovascular** |  |  |  |  |  |  |
| No of cases | 1,356 | 1,160 | 1,165 | 1,171 | 1,486 |  |
| Age-adjusted HR  (95% CI) | 1.00 | 0.85  (0.79, 0.92) | 0.90  (0.84, 0.98) | 0.82  (0.76, 0.89) | 0.91  (0.84, 0.98) | 0.061 |
| Multivariable-adjusted HR  (95% CI) | 1.00 | 0.95  (0.87, 1.03) | 1.01  (0.93, 1.11) | 0.94  (0.86, 1.04) | 0.98  (0.88, 1.08) | 0.810 |
| **Cancer** |  |  |  |  |  |  |
| No of cases | 1,067 | 1,044 | 1,003 | 1,073 | 1,234 |  |
| Age-adjusted HR  (95% CI) | 1.00 | 0.94  (0.87, 1.03) | 0.92  (0.85, 1.00) | 0.92  (0.85, 1.01) | 0.97  (0.89, 1.05) | 0.663 |
| Multivariable-adjusted HR  (95% CI) | 1.00 | 0.99  (0.90, 1.09) | 0.99  (0.89, 1.09) | 1.00  (0.91, 1.11) | 1.02  (0.92, 1.14) | 0.528 |
| Adjusted for attained age (years), gender, education level (≤12 or >12 y), waist circumference (<80, 80-87, ≥88 cm), hypertension (yes/no), hypercholesterolemia (yes/no), weight loss >5kg within 1 year (yes/no), leisure-time inactivity (≤2 hours/day or >2 hours per day) and daily walking/cycling (≤40 minutes/day or >40 minutes/day), family history of myocardial infarction before the age of 60 years (yes/no), smoking status (current, former, never), use of aspirin (yes/no), energy intake (continuous, kcal/day), Mediterranean diet (9-score), parity (0, 1-2, ≥ 3 child), use of hormone replacement therapy (yes/no) and dietary PCB exposure (quintiles), EPA-DHA intake (quintiles) | | | | | | |
